# Case of Poisoning

**Published:** 1862-03

**Authors:** A. K. Van Horn

**Affiliations:** Baileyville, Ill.


### CASE OF POISONING.

By A. K. VAN HORN, M. D., of Baileyville, Ill.

On September 4th, 1861, S. Walters requested me to visit his daughter, a child about four years old, who, he said, "had been taken with fits," a short time previous. I learned on my arrival, that she had commenced to "act strangely" very soon after her return from a grove, where she had spent the morning looking for berries. Her mother, alarmed at the symptoms, had given a large table spoonful of castor oil, which produced no effect, beyond a slight emesis at the moment of administration. She had continued to grow worse for an hour after reaching home, but since then had remained in much the same condition, in which I found her, on my arrival, two and a half hours after the commencement of her attack. Her symptoms were so peculiar and interesting that I venture to describe them at some length.

Her general appearance indicated good health, and to a casual observer would have presented no very unusual feature, except the fixed, expressionless eye. She uttered no complaint, and except the slight nausea, caused by the offensive taste of the oil, appeared to have had no sickness at her stomach. Her hands, feet, and face were considerably swollen, and hot to the touch; her *face, redder* than usual; her body was a little above the natural temperature, dry, and on the more delicate parts a slight scarlet rash was visible; the abdomen was hard, with slight meteorism; the pulse was a little accelerated and feeble; the tongue was not much coated, but very dry; the mouth and fauces were parched, as if with thirst, and of a dusty, red color. There appeared to be unpleasant sensations in the mouth and larynx, as the patient frequently seemed as if trying to rub her tongue with her fingers, and often clutched at her throat.

But it was on the *nervous system* that the force of the poison mainly spent itself. That part of it, which guides and governs the voluntary motions, producing the harmony of action so necessary to happiness, and even existence, seemed to have resigned its seat to a tricky elf, who revelled in the mad disorder he created among the muscles, and who, touching the wrong keys, like an unskillful player, got not *music*, but *discord*, from the "harp of a thousand strings." Even the external senses, sight, hearing, &c., did not escape the mad spirit's fingers, who filled the "porches of the ears" with strange sound, and led whole troops of phantoms to vex the fixed eye.

She lost the power of locomotion almost entirely, and stood with the air and attitude of one deeply intoxicated. The slightest inclination from the perpendicular resulted in a fall. If assisted to walk, she staggered along, with the body bent forward. Every few minutes she would have an attack of convulsions, affecting principally her legs and arms, and rapid and violent as though caused by an electric shock, but apparently painless. The hearing seemed almost closed to external sounds; but unreal ones were present, as she sometimes inquired, "what made that big noise?" when the room was entirely still. The faculty of speech, though not *lost*, was so changed that much of what she said was unintelligible. Among all the organs, none exhibited so marked peculiarity as her eyes! The pupils were enormously dilated, seeming to cover almost the whole front of the globe, and insensible to light. Unreal objects flitted before them, which she was always endeavoring to catch; but sight was not entirely abollshed, as she would grasp at articles presented to her; but she frequently reached too far, or not far enough, as if unable to appreciate distance correctly. Nor were the emotions unaffected. She talked almost incessantly. After a few moments of silence, she would burst into a violent fit of weeping, which often ended in a paroxysm of boisterous laughter, and thus she swung, "a pendulum betwixt a smile and tear."

In about six hours from the commencement of the attack the symptoms began to decline in violence; her hearing grew more acute, face less flushed, and the convulsions less marked, and recurring at longer intervals. During the night following she slept profoundly, and the next day had no symptoms, save slight vertigo and dilated pupils, which passed off in a short time, leaving to the patient her usual health.

The usual treatment by emetics and cathartics was adopted. There is no known antidole to this class of poisons. Very large doses were required to produce any effect, as the stomach and bowels seemed paralyzed and impassive to the amounts ordinarily administered. No traces of the poison were seen in the evacuations. Large quantities of stramonium were found growing near the house, and in the grove, where she had been wandering, many stalks of a plant answering to the description of belladona were found, each full of its beautiful, but treacherous red berries.

Both of these poisons belong to the class called by Wood, *Cerebral Stimulants*, and have many symptoms in common; but from a consideration of all the symptoms, I incline to the belief that the child had eaten berries gathered from the Deadly Nightshade.

